# Supplementary material for: Non-Structural Protein-W61 as a Novel Target in Severe Fever with Thrombocytopenia Syndrome Virus (SFTSV): An In-Vitro and In-Silico Study on Protein-Protein Interactions with Nucleoprotein and Viral Replication
Source: Viruses. 2023 Sep 20;15(9):1963. doi: 10.3390/v15091963 (PMC10535573; doi:10.3390/v15091963)
Supplement: Supplementary file 1 [file viruses-15-01963-s001.zip › proof reading-Supplementary data_viruses.pdf]

Supplementary information

Non-Structural Protein-W61 as a Novel Target in Severe Fever with Thrombocytopenia Syndrome Virus (SFTSV): An In-Vitro and In-Silico Study on Protein-Protein Interactions with Nucleoprotein and Viral Replication

Supplementary Table S1. Bacterial strains and plasmids use in this study

Strains/plasmid	Description	References
Escherichia coli		
DH5α	F^{-endA1}, glnV44, thi-1, recA1, relA1, gyrA96, deoR, nupG, Φ80dlacZΔM15 Δ(lacZYA-argF)U169, hsdR17(rK-mK^{+}),	Lab stock
BL21(DE3)pLysS	F^{-}, ompT, hsdS_B (r_B–, m_B^{-}), dcm, gal, λ (DE3), pLysS, Cm^r	Lab stock
JOL2643	DH5α containing pcDNA3.1-NP	This study
JOL2644	DH5α containing pcDNA3.1-NSs	
JOL2645	DH5α containing pcDNA3.1-NSsC5S	
JOL2646	DH5α containing pcDNA3.1-NSsW61Y	
JOL2647	DH5α containing pcDNA3.1-NSsS207T	
**Plasmids**		
pcDNA3.1-CMV	CMV promoter, MCS, f1 ori, SV40 promoter, Neomycinr^{R} pUC ori, Amp^{R}	Lab stock
pcDNA3.1-NP	pcDNA3.1 harboring NP of SFTSV	This study
pcDNA3.1-NSs	pcDNA3.1 harboring NSs of SFTSV	
pcDNA3.1-NSsC5S	pcDNA3.1 harboring NSC5S mutant of NSs	
pcDNA3.1- NSsW61Y	pcDNA3.1 harboring NSW61Y mutant of NSs	
pcDNA3.1- NSsS207T	pcDNA3.1 harboring NSS207T mutant of NSs	
pET28a(+)	IPTG-inducible, T7 expression vector, C-terminal 6× histidine tag, Kan^{R}	Lab stock
pET28a-NP	pET28a(+)harboring NP optimized genes	This study
pET28a-NSs	pET28a(+)harboring NSs genes	This study
pET28a-NSsW61Y	pET28a(+)harboring NSs W61Y genes	This study

Supplementary Table S2. Primers use in this study

primers	Description (5' - 3')	References
pcDNA3.1-NP-F	CCCAAGCTTGCCACCATGGCAGAGT	
pcDNA3.1-NP-R	CGGAATTCTTACAGGTTTCTGTAAGCAGCA	
pcDNA3.1- NSs-F	CCCAAGCTTATGTCGCTGAGCAA	
pcDNA3.1-NSs-R	CGAAGCTTTTAGACCTCCTTCG	This study
pET28-NSs-F	CGGAATTCATGTCGCTGAGCAAATGCT	
pET28-NSs-R	GGGAAGCTTGTGGTGGTGGTGG	
pET28-NP-F	CGGAATTCATGTCCGAGTGGAGC	
pET28-NP-R	GGGAAGCTTCAGGTTCCGGTAAG	
Sseg-F-3	GGGTCCCTGAAGGAGTTGTAAA	Y. Sun et al. Journal of Clinical Virology 53 (2012) 48–53
Sseg-R-3	TGCCTTCACCAAGACTATCAATGT	
Site-directed mutagenesis		
NSst16a-F	ATGTCGCTGAGCAAAAGCTCCAACGTTGACCTCA AATC	This study
NSst16a-R	GATTTGAGGTCAACGTTGGAGCTTTTGCTCAGCGA CA	
NSsg182a-g183t-F	GGATTTTTTGGGTTGCCAAATAAATATACTGTGTTG GTCATCTTGCCC	
NSsg182a-g183t-R	GGGCAAGATGACCAACACAGTATATTTATTTGGCA ACCCAAAAAATCC	
NSst619a-F	CTTGAACCACCACTTATTCACTTCTACCTCATTGCG TAAG	
NSst619a-R	CTTACGCAATGAGGTAGAAGTGAATAAGTGGTGGT TCAAG	

Supplementary Table S3. Pairwise comparison of nucleotide sequence alignment of complementary sequences within S segment of completely sequenced twelve Korean SFTSV isolates belonging to different genotypes. The lower comparison gradient indicated the percentage identity between two strains. Pairwise comparison was performed using CLC genomics workbench 22 software.

S segment	1	2	3	4	5	6	7	8	9	10	11	12
CB2												
CB1	95.30											
CB3	95.02	96.45										
KADGH	96.11	96.11	96.05									
KAGNH	95.82	95.93	96.22	96.02								
KAGWH3	95.76	96.16	95.88	96.68	97.37							
KAGNH4	96.33	96.56	96.51	97.37	97.94	98.28						
KAJNH2	95.36	95.59	95.53	97.77	95.88	96.28	96.79					
KAGBH5	95.70	96.11	95.93	96.74	97.54	99.03	98.45	96.51				
KAGBH6	95.70	96.33	95.59	96.39	97.31	98.22	98.00	95.99	98.17			
KACNH3	95.07	96.74	98.00	96.39	96.22	95.99	96.68	95.76	96.05	95.82		
KASJH	95.99	94.96	94.79	95.48	95.48	95.76	96.33	94.79	95.88	95.65	94.79	

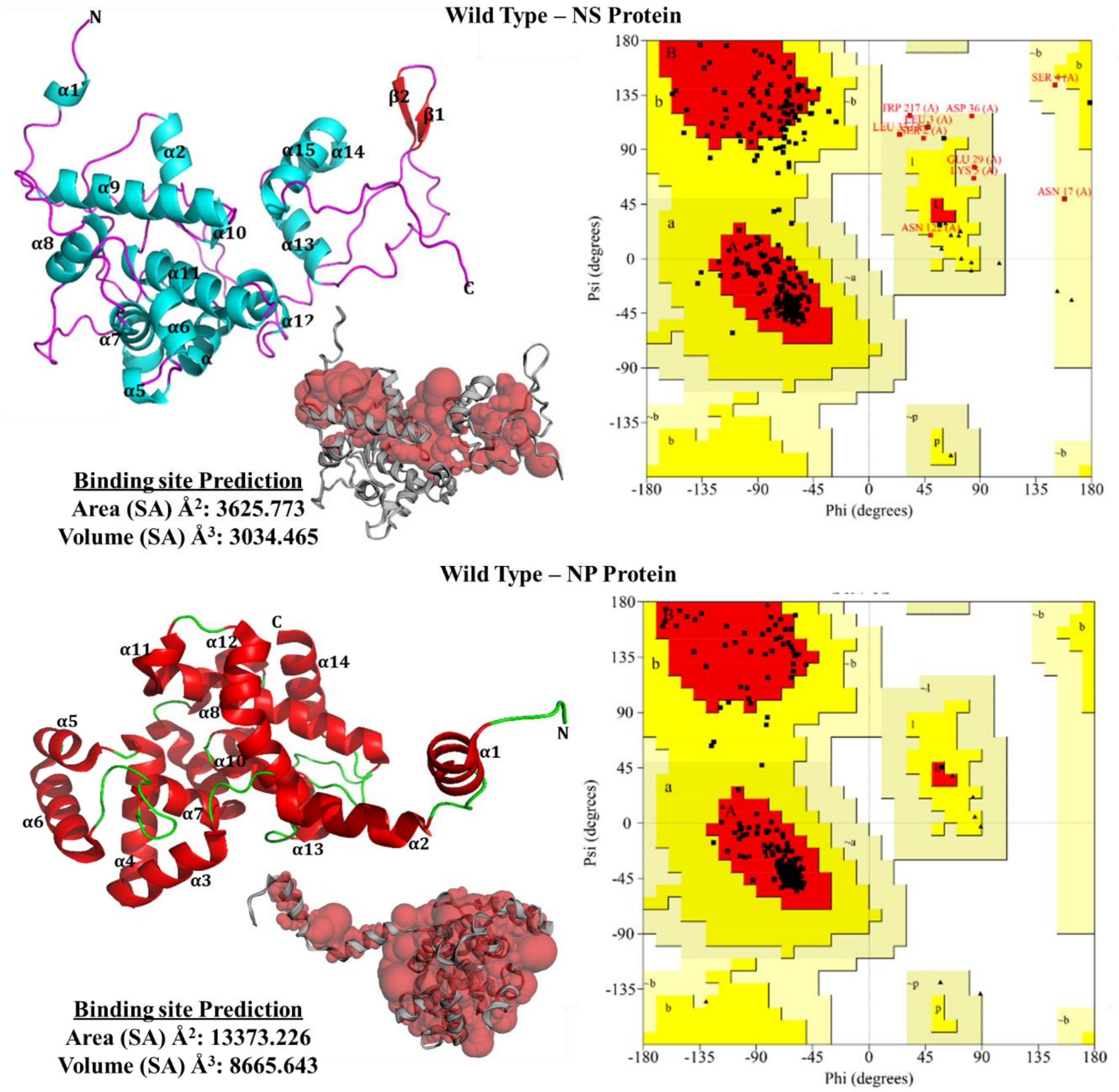

Supplementary Figure S1. Prediction of the 3D structure and Ramachandran plot analysis of the NSs and NP protein. The 3D structure of NSs and NP protein was modelled using AlphaFold and performed quality verification using the PROCHECK program. Ramachandran Plot analysis of the refined model of the NSs and NP proteins showing the number of residues located in a favourable region.

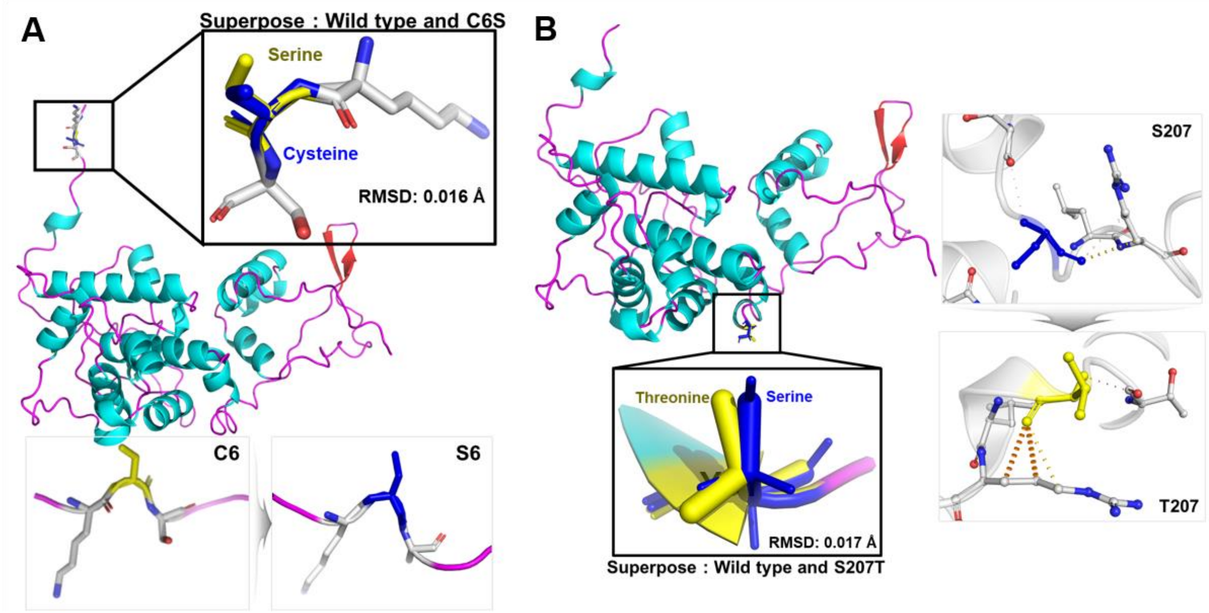

Supplementary Figure S2. Prediction of the NSs mutants docking structure (A) Molecular docking of NSsC6S with NP and (B) Molecular docking of NSsS207T with NP.

Supplementary Figure S3. Alignment of Consensus Sequences of the S segments from SFTSV strains

Supplementary Figure S4. Alignment of Consensus Sequences of the M segments from SFTSV strains
