# Supplementary figures and images for: Non-Structural Protein-W61 as a Novel Target in Severe Fever with Thrombocytopenia Syndrome Virus (SFTSV): An In-Vitro and In-Silico Study on Protein-Protein Interactions with Nucleoprotein and Viral Replication
Source: Viruses. 2023 Sep 20;15(9):1963. doi: 10.3390/v15091963 (PMC10535573; doi:10.3390/v15091963)

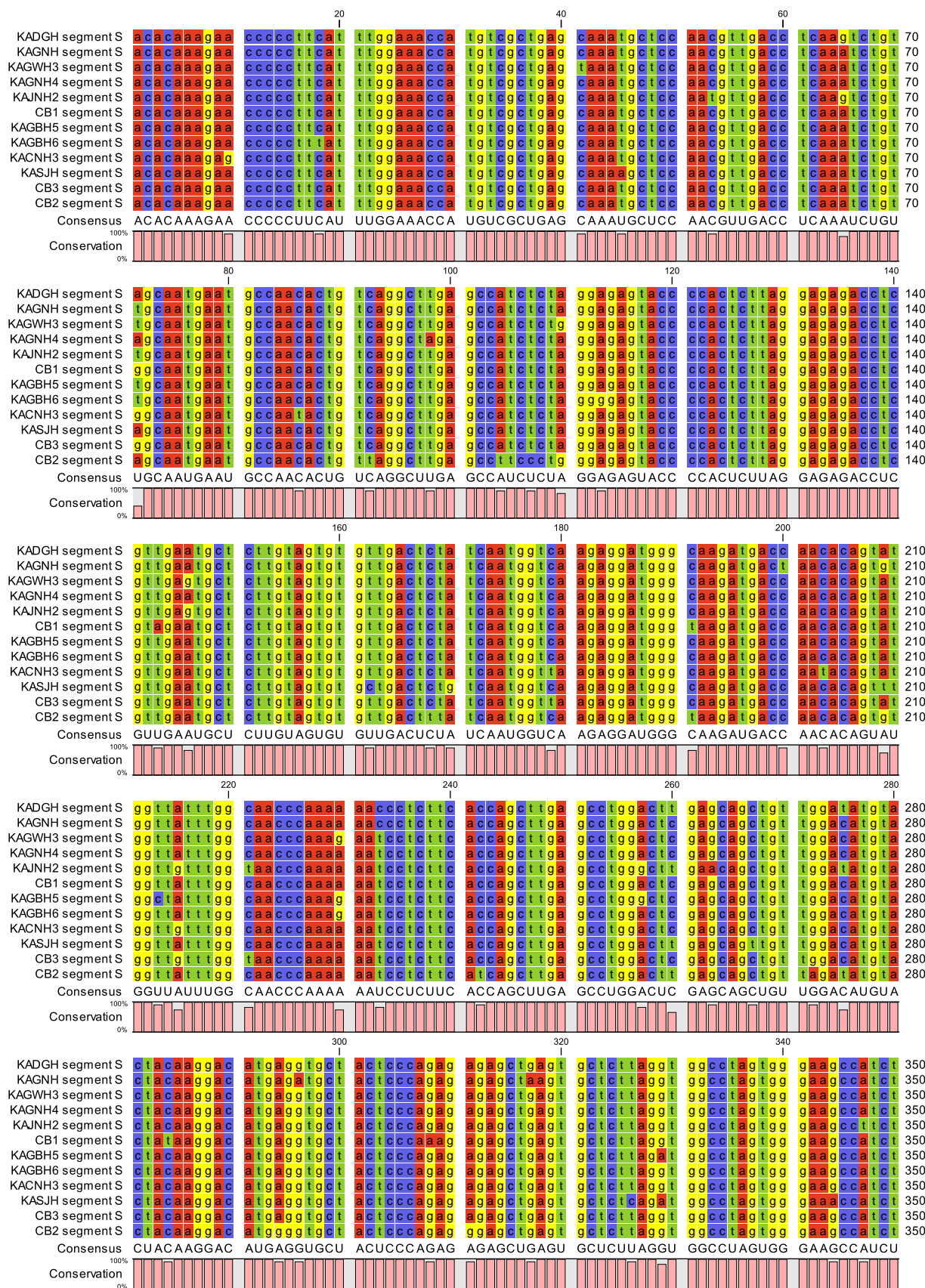

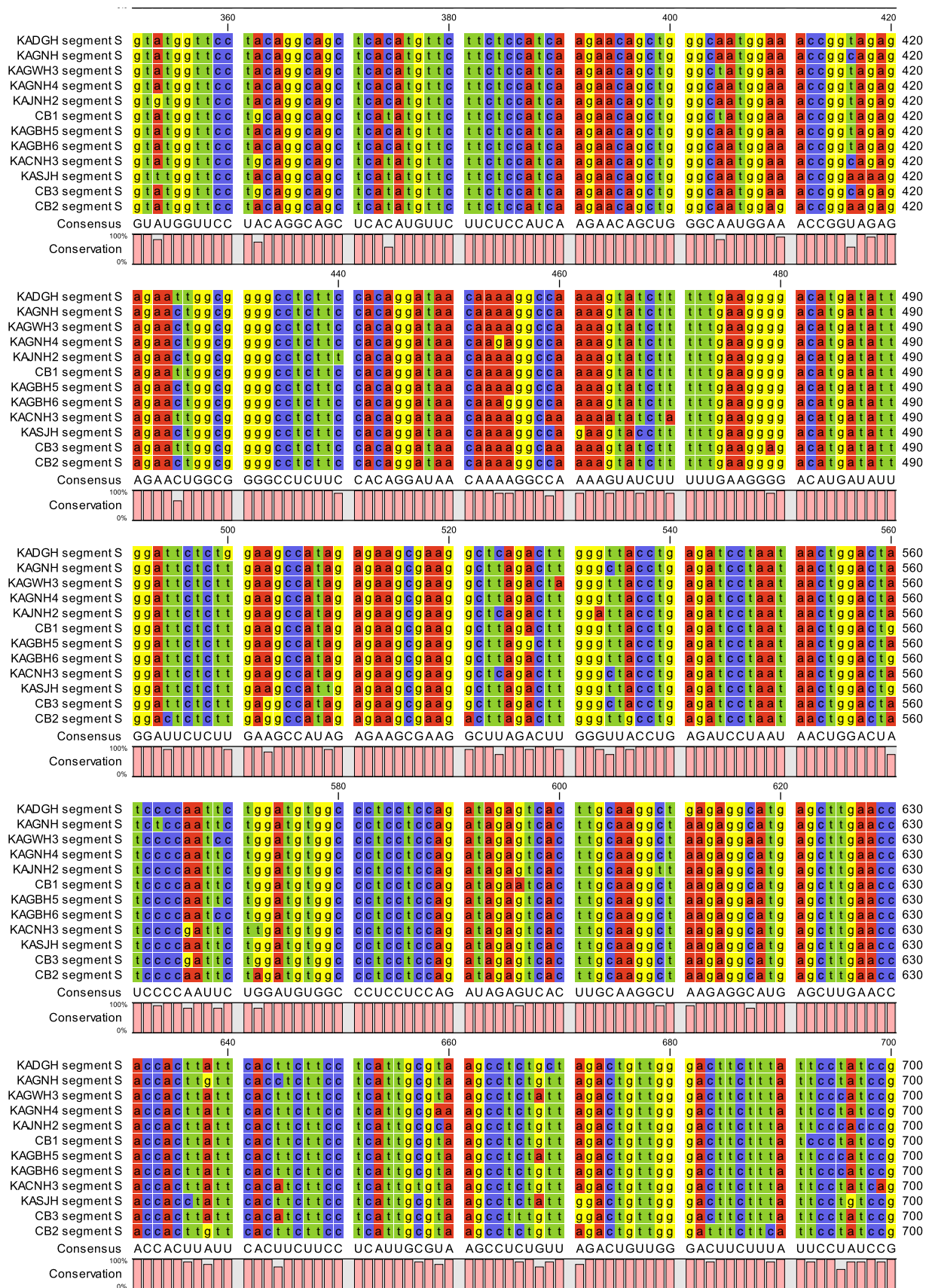

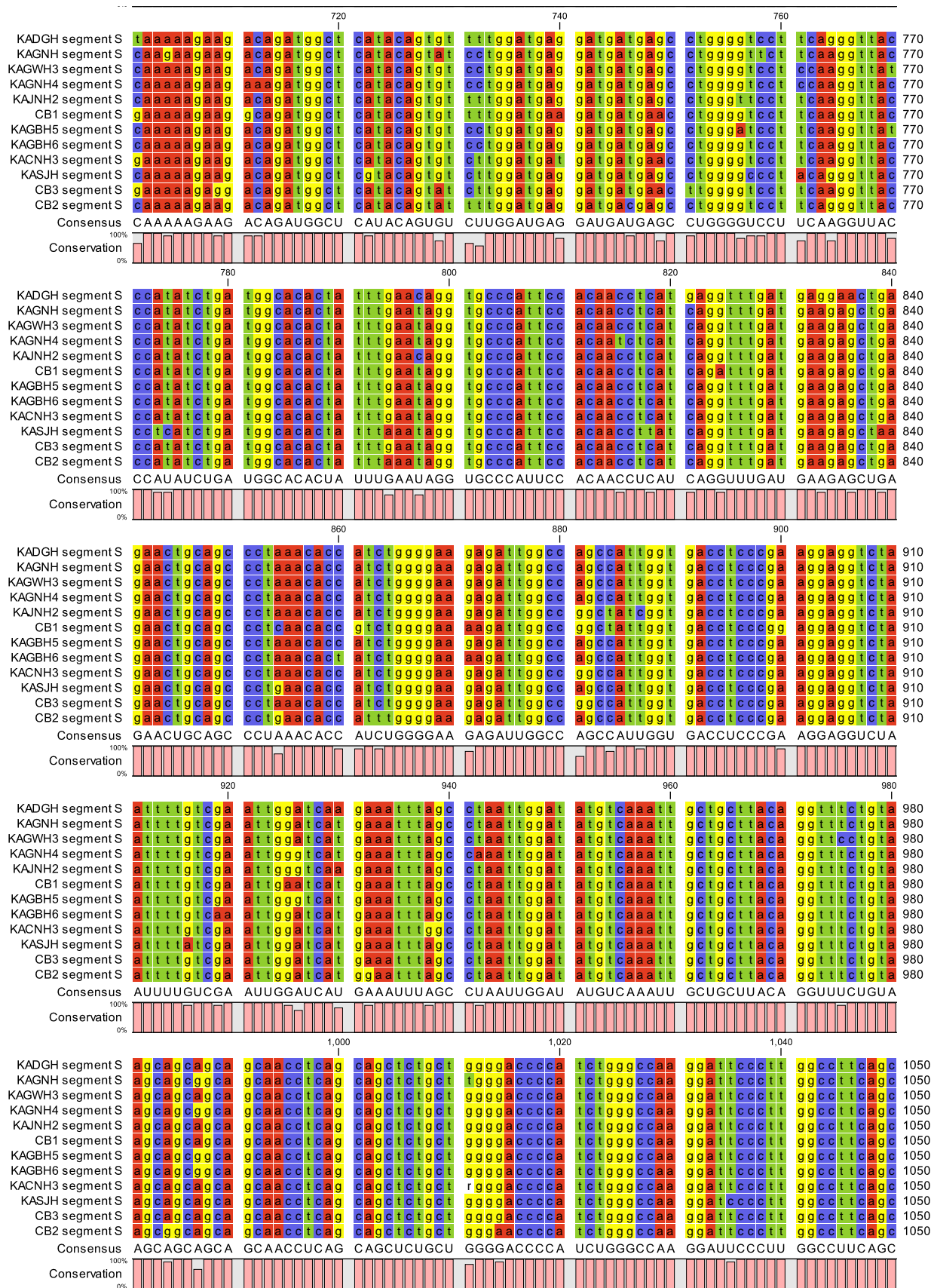

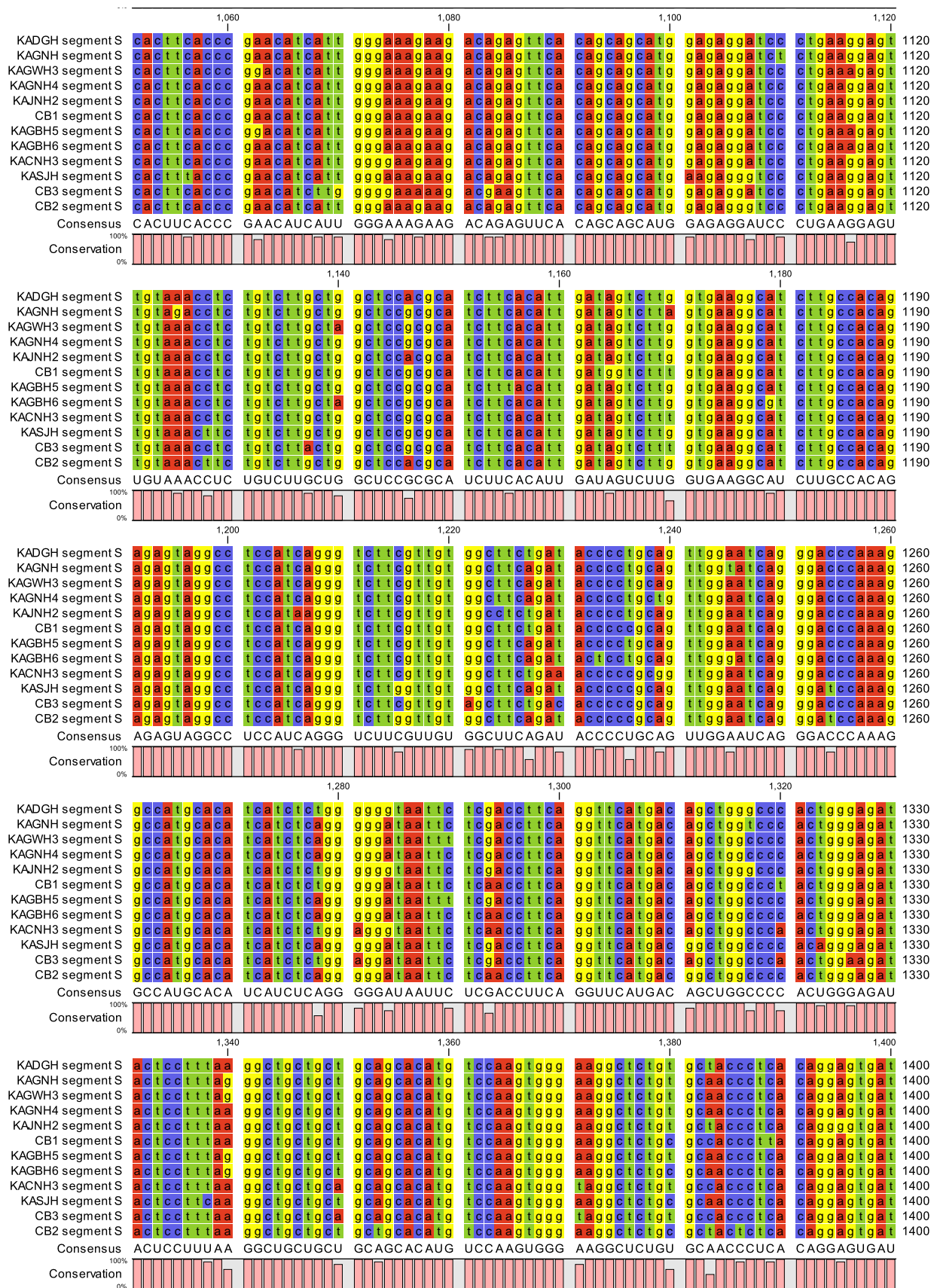

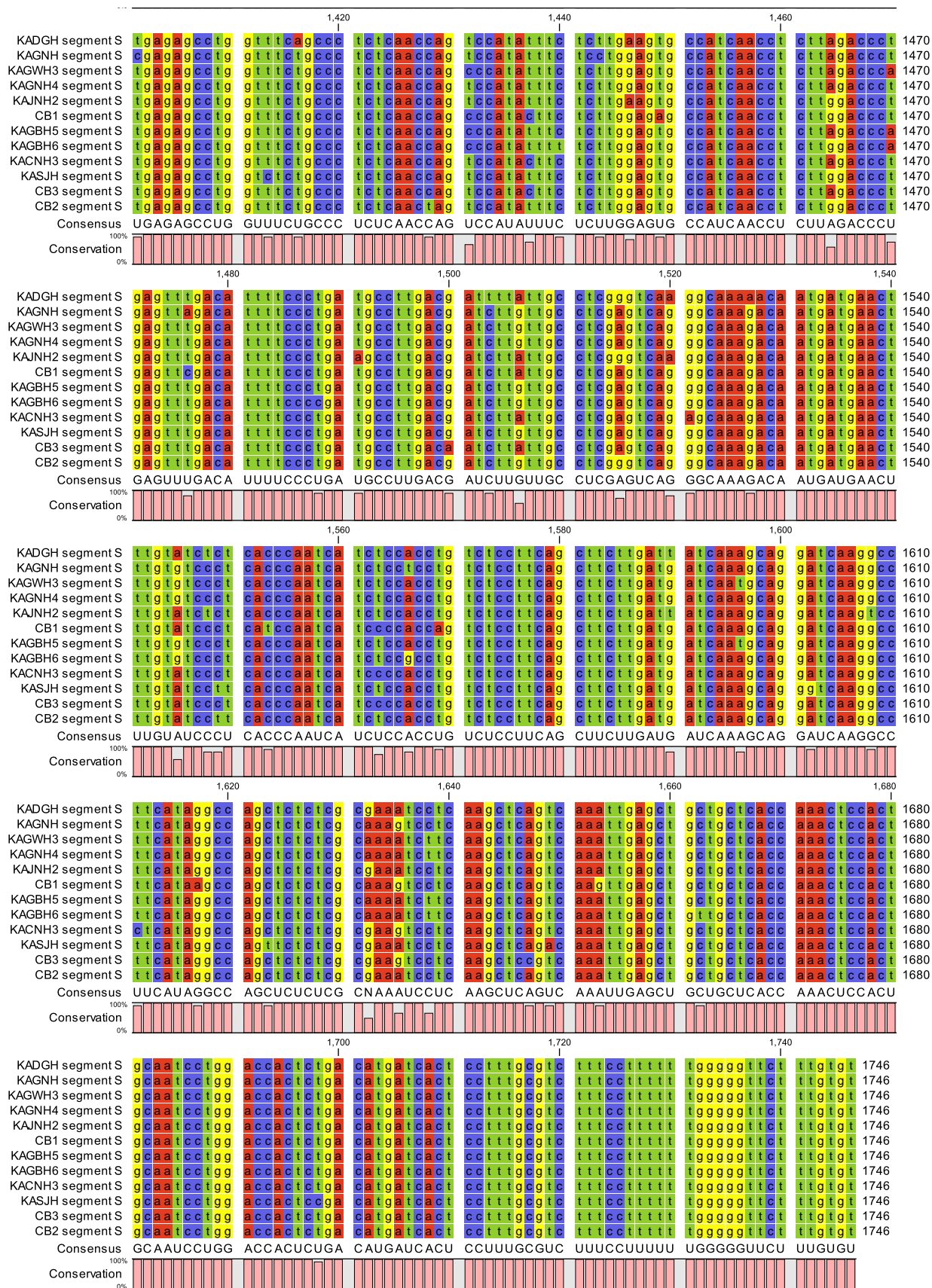

Supplement: Supplementary file 1 [file viruses-15-01963-s001.zip › Supplementary Figure S3- Alignment of Consensus Sequences of the S segments from SFTSV strains.pdf]

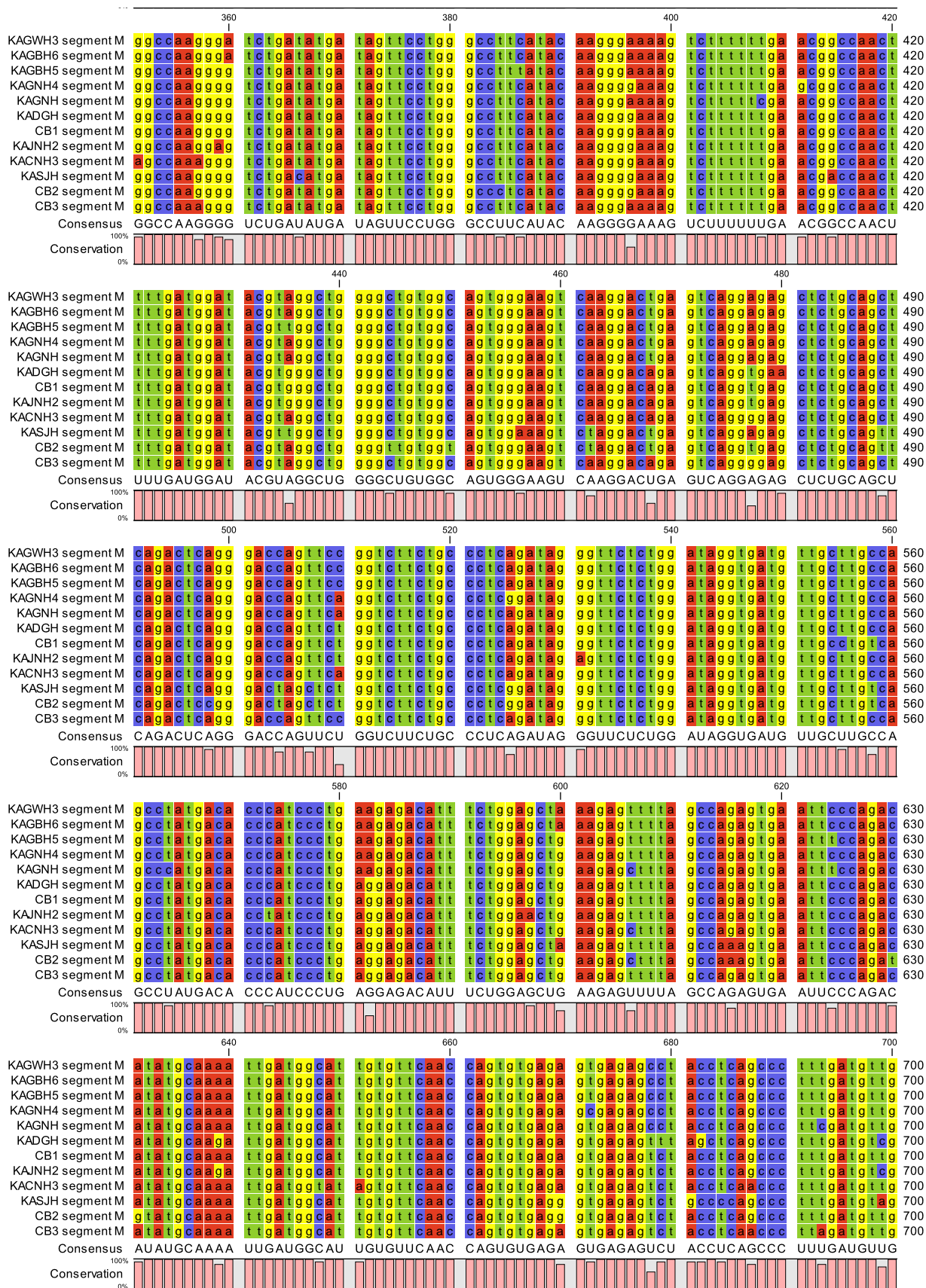

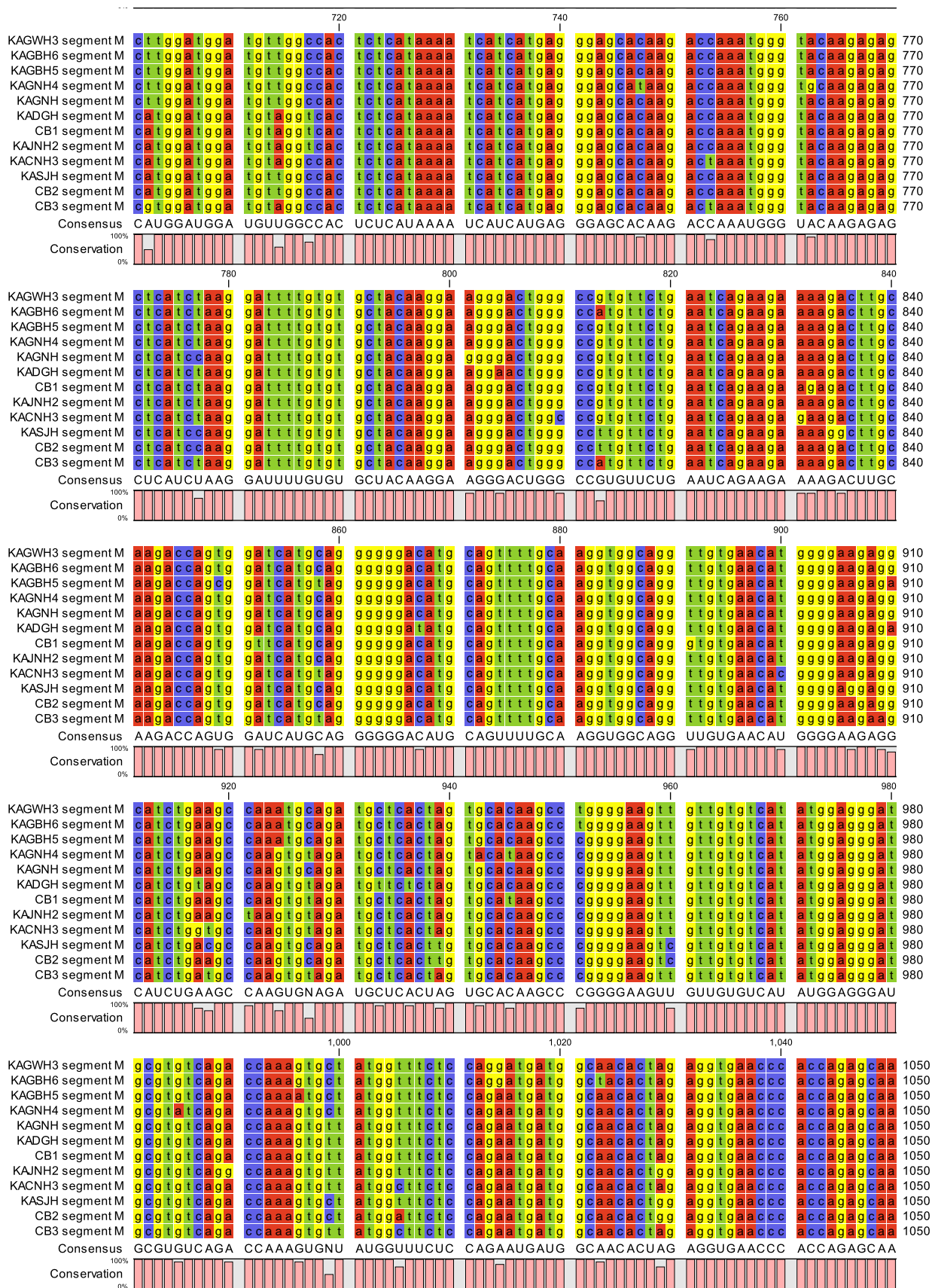

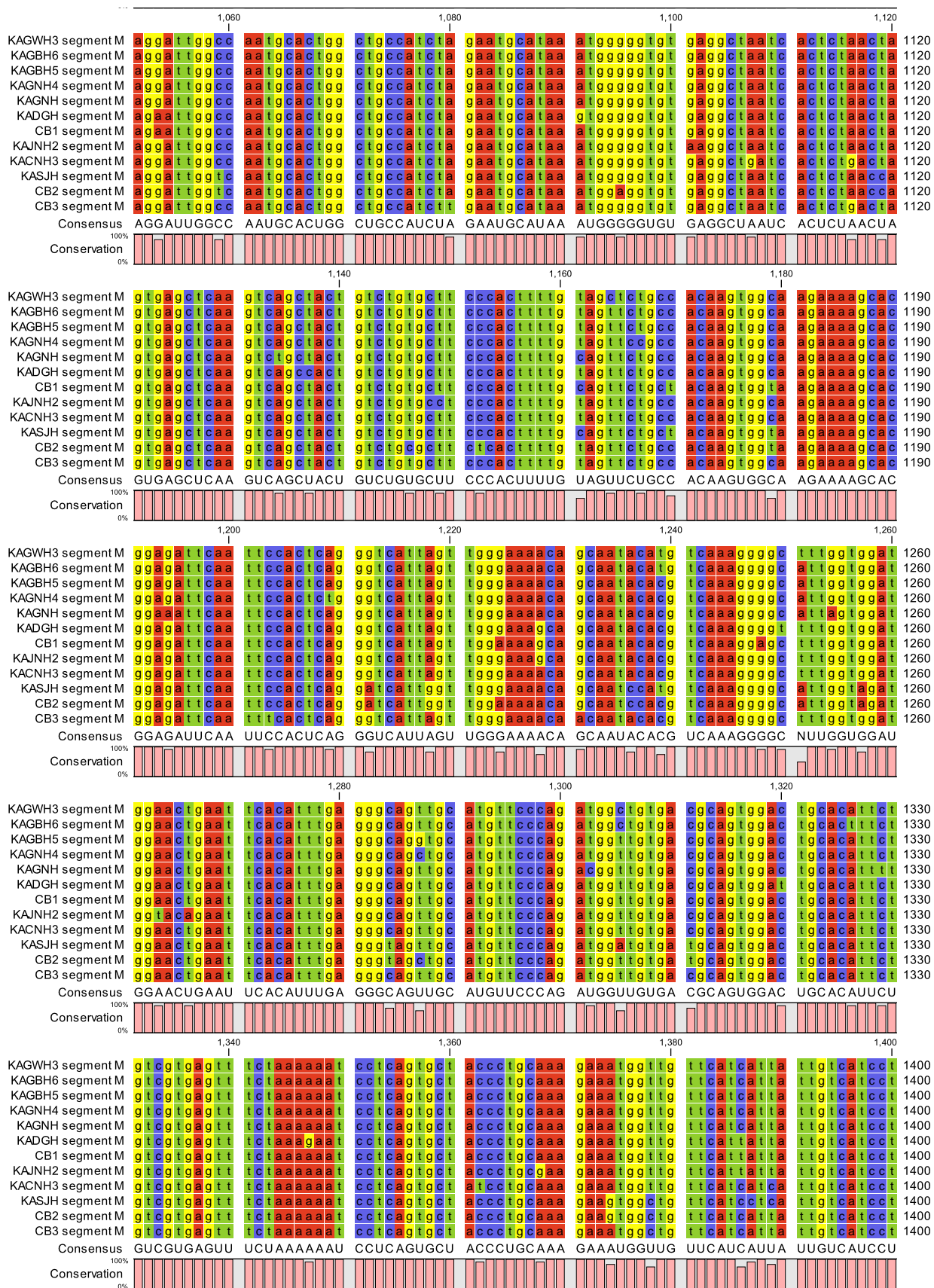

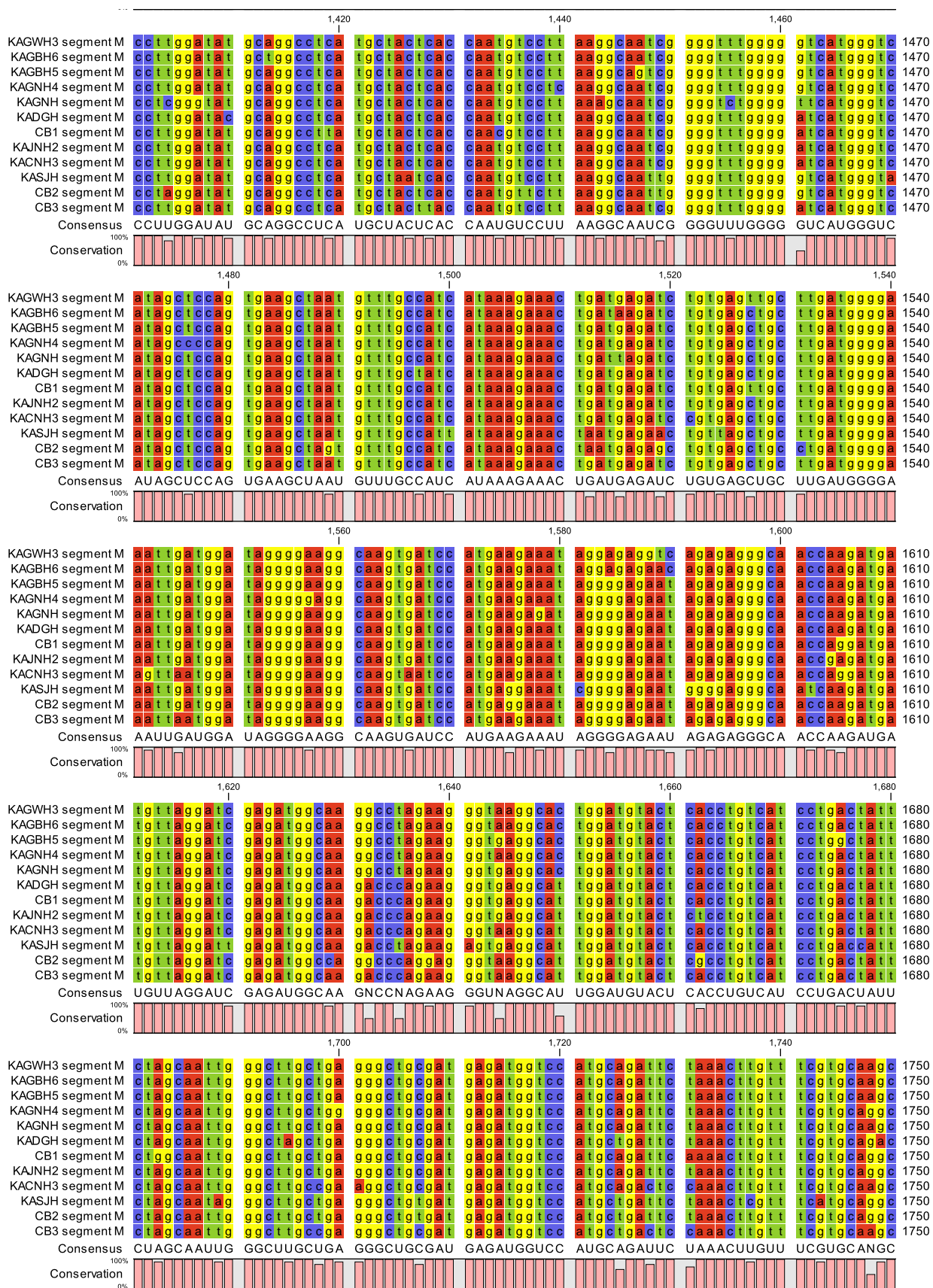

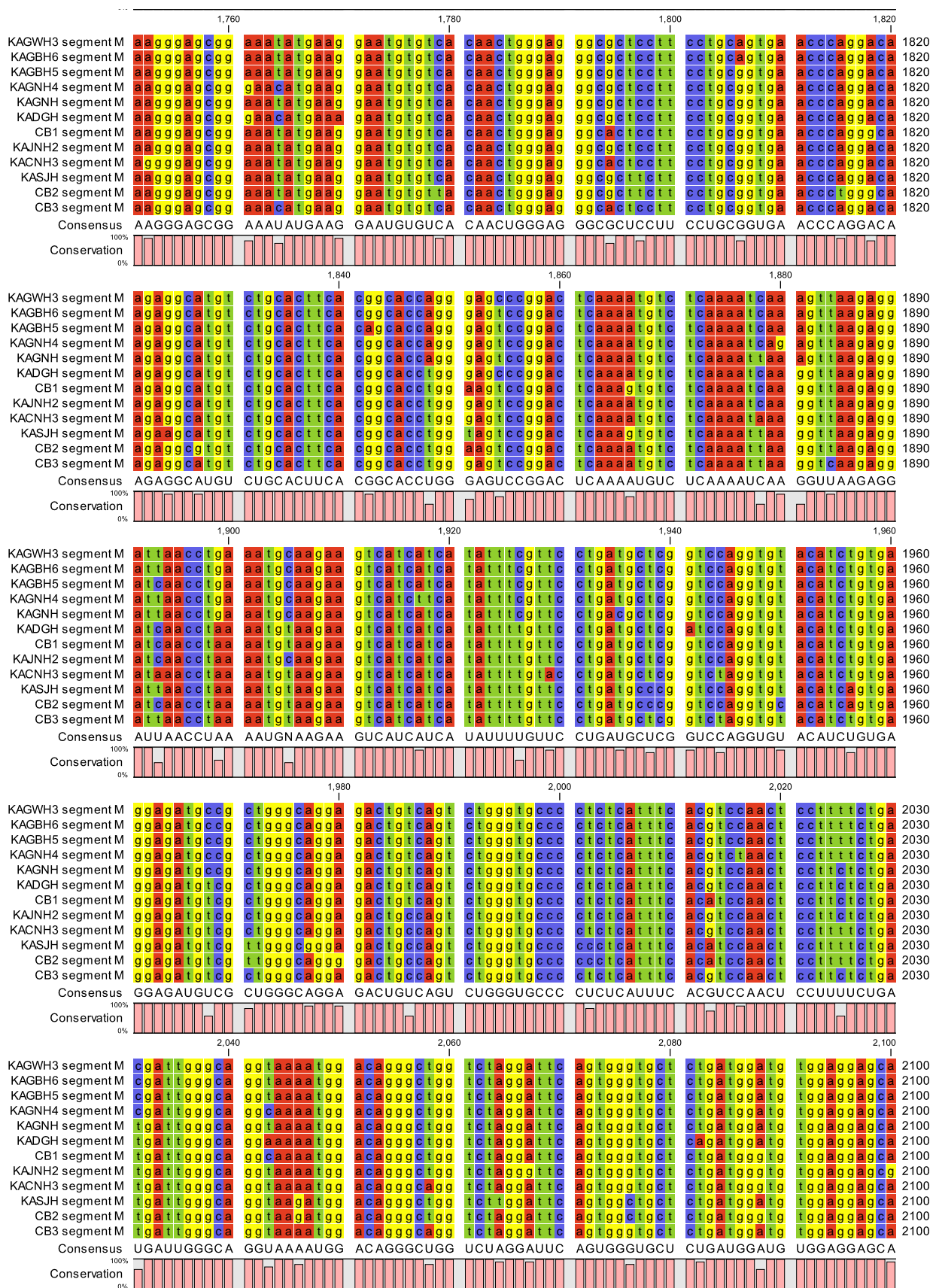

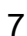

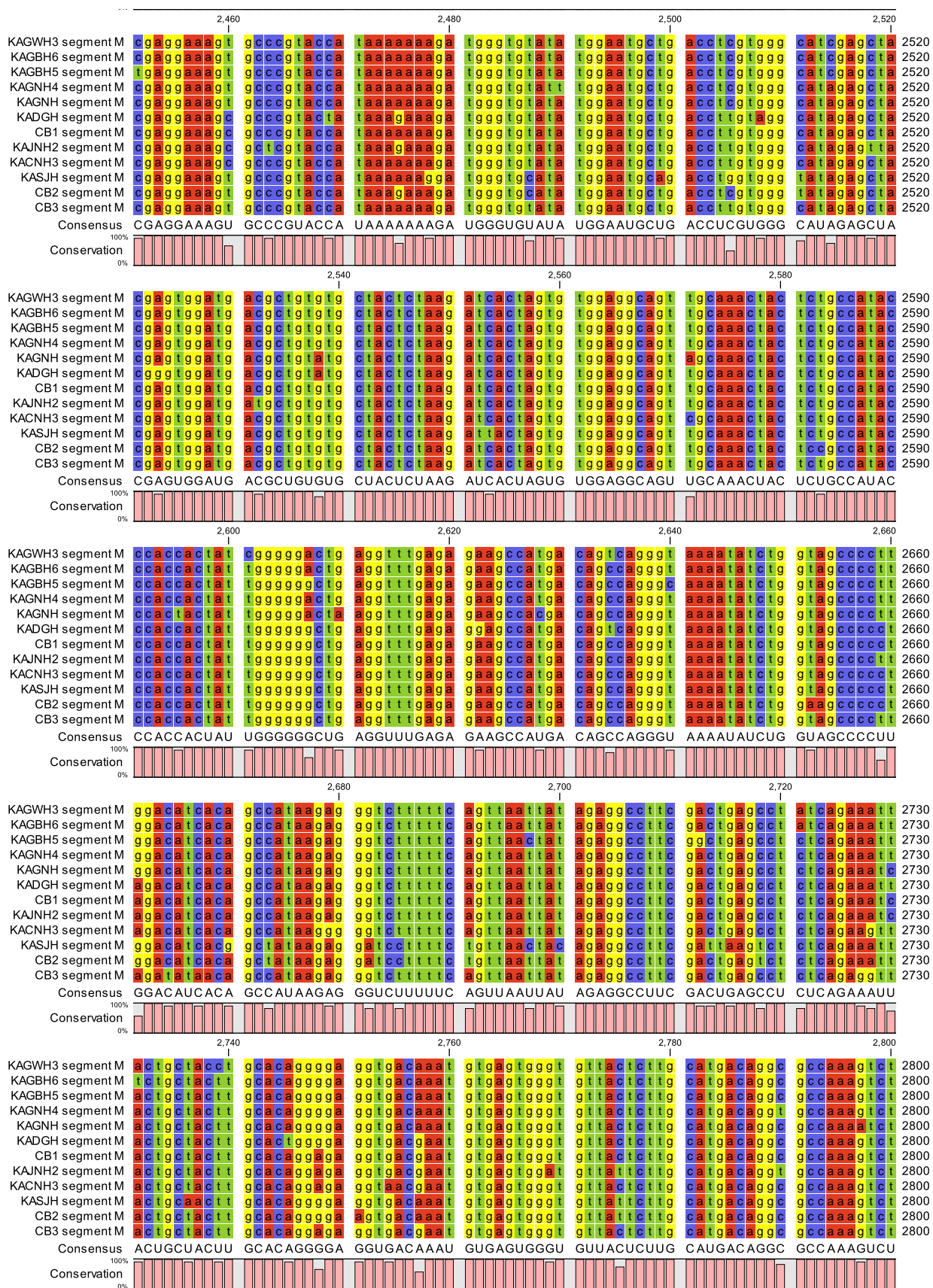

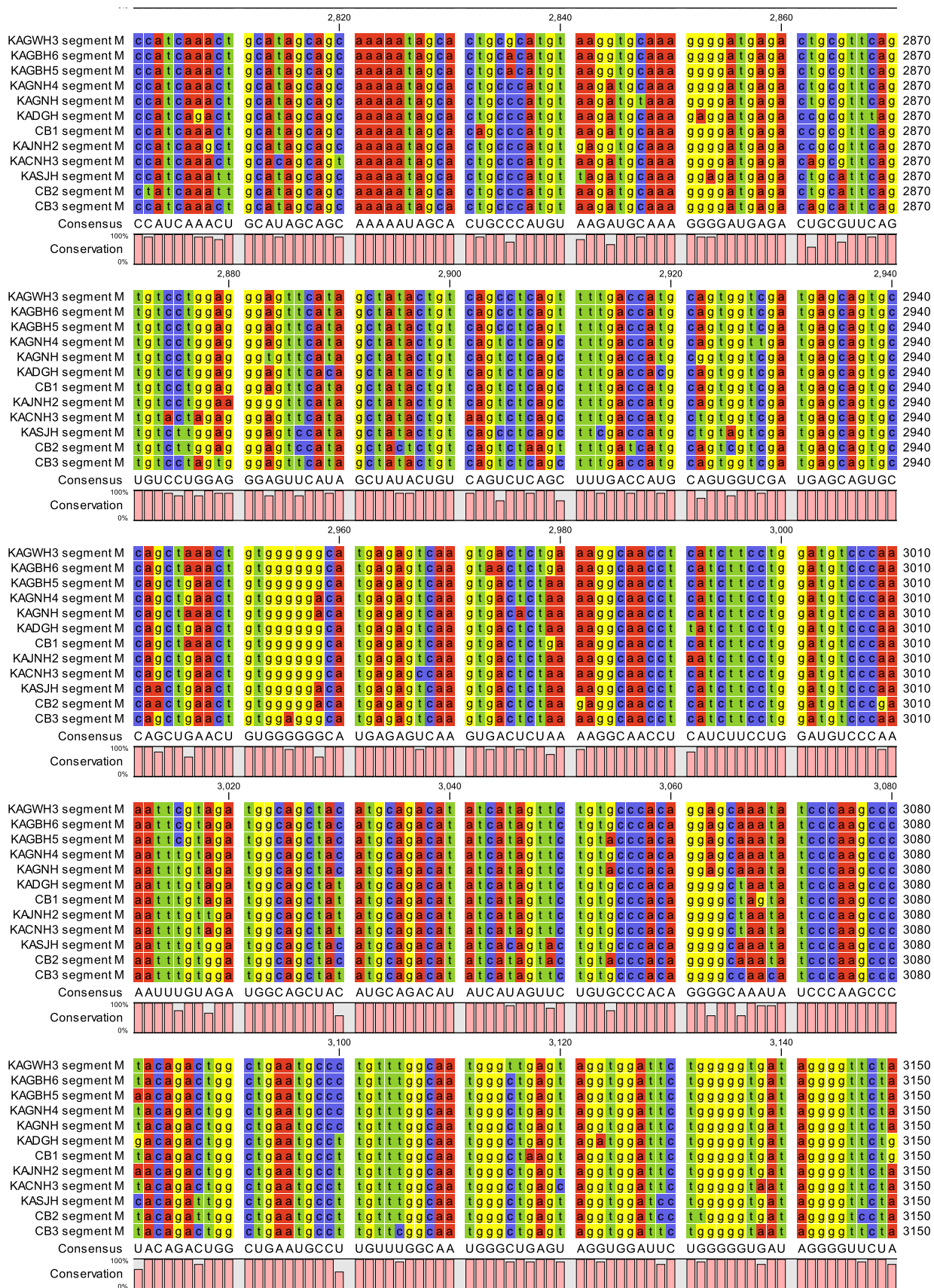

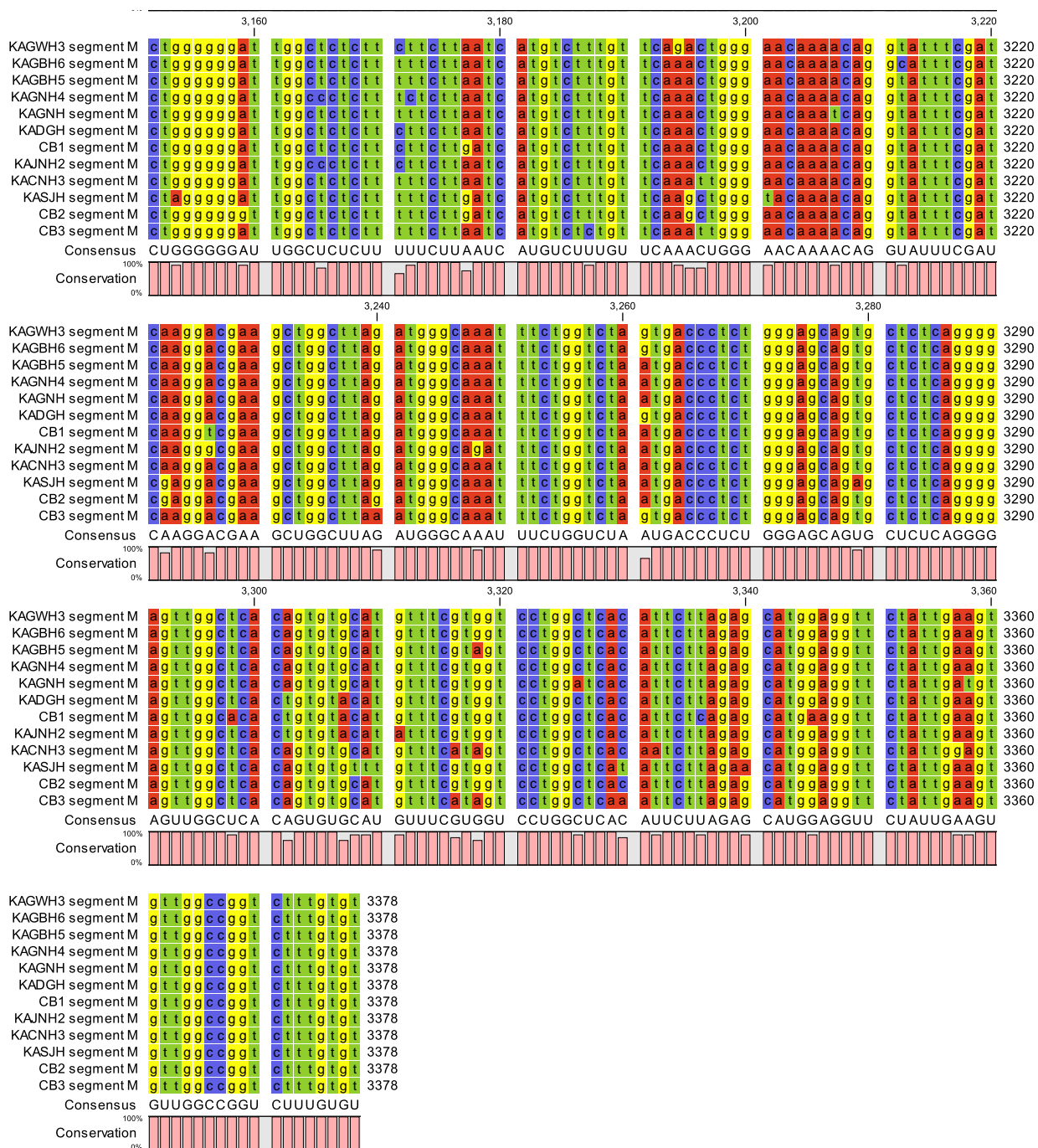

Supplement: Supplementary file 1 [file viruses-15-01963-s001.zip › Supplementary Figure S4- Alignment of Consensus Sequences of the M segments from SFTSV strains.pdf]
